# Membrane transporters in the bioproduction of organic acids: state of the art and future perspectives for industrial applications

**DOI:** 10.1093/femsle/fnaa118

**Published:** 2020-07-18

**Authors:** I Soares-Silva, D Ribas, M Sousa-Silva, J Azevedo-Silva, T Rendulić, M Casal

**Affiliations:** Centre of Molecular and Environmental Biology (CBMA), Department of Biology, University of Minho, Campus de Gualtar, Braga 4710-057, Portugal; Institute of Science and Innovation for Bio-Sustainability (IB-S), University of Minho, Campus de Gualtar, Braga 4710-057, Portugal; Centre of Molecular and Environmental Biology (CBMA), Department of Biology, University of Minho, Campus de Gualtar, Braga 4710-057, Portugal; Institute of Science and Innovation for Bio-Sustainability (IB-S), University of Minho, Campus de Gualtar, Braga 4710-057, Portugal; Centre of Molecular and Environmental Biology (CBMA), Department of Biology, University of Minho, Campus de Gualtar, Braga 4710-057, Portugal; Institute of Science and Innovation for Bio-Sustainability (IB-S), University of Minho, Campus de Gualtar, Braga 4710-057, Portugal; Centre of Molecular and Environmental Biology (CBMA), Department of Biology, University of Minho, Campus de Gualtar, Braga 4710-057, Portugal; Institute of Science and Innovation for Bio-Sustainability (IB-S), University of Minho, Campus de Gualtar, Braga 4710-057, Portugal; Centre of Molecular and Environmental Biology (CBMA), Department of Biology, University of Minho, Campus de Gualtar, Braga 4710-057, Portugal; Institute of Science and Innovation for Bio-Sustainability (IB-S), University of Minho, Campus de Gualtar, Braga 4710-057, Portugal; Centre of Molecular and Environmental Biology (CBMA), Department of Biology, University of Minho, Campus de Gualtar, Braga 4710-057, Portugal; Institute of Science and Innovation for Bio-Sustainability (IB-S), University of Minho, Campus de Gualtar, Braga 4710-057, Portugal

**Keywords:** industrial biotechnology, cell factories, carboxylic acids, transporter proteins, permease

## Abstract

Organic acids such as monocarboxylic acids, dicarboxylic acids or even more complex molecules such as sugar acids, have displayed great applicability in the industry as these compounds are used as platform chemicals for polymer, food, agricultural and pharmaceutical sectors. Chemical synthesis of these compounds from petroleum derivatives is currently their major source of production. However, increasing environmental concerns have prompted the production of organic acids by microorganisms. The current trend is the exploitation of industrial biowastes to sustain microbial cell growth and valorize biomass conversion into organic acids. One of the major bottlenecks for the efficient and cost-effective bioproduction is the export of organic acids through the microbial plasma membrane. Membrane transporter proteins are crucial elements for the optimization of substrate import and final product export. Several transporters have been expressed in organic acid-producing species, resulting in increased final product titers in the extracellular medium and higher productivity levels. In this review, the state of the art of plasma membrane transport of organic acids is presented, along with the implications for industrial biotechnology.

## Teaser

Transporter protein expression enables the optimization of microbial cell factories for the bioproduction of organic acids.

## INTRODUCTION

Organic acids are an essential group of platform chemicals produced by microbes. Most of the organic acids produced industrially are used in the food industry. Currently, the major source of production of these compounds is the chemical synthesis from petroleum derivatives. Nonetheless, several organic acids are already industrially generated *via* microbial cell-factories, including succinic, lactic, citric, gluconic and acetic acid (Alonso, Rendueles and Díaz 2015). Microbial production of organic acids comprises several membrane transport processes, mostly controlled by membrane proteins, namely substrate import, transport of metabolites between organelles and product export. These processes, critical for the bioproduction of organic acids, are the major topic of this review.

## MICROBIAL CELL FACTORIES IN THE PRODUCTION OF BIO-BASED ORGANIC ACIDS

**Figure 1.**
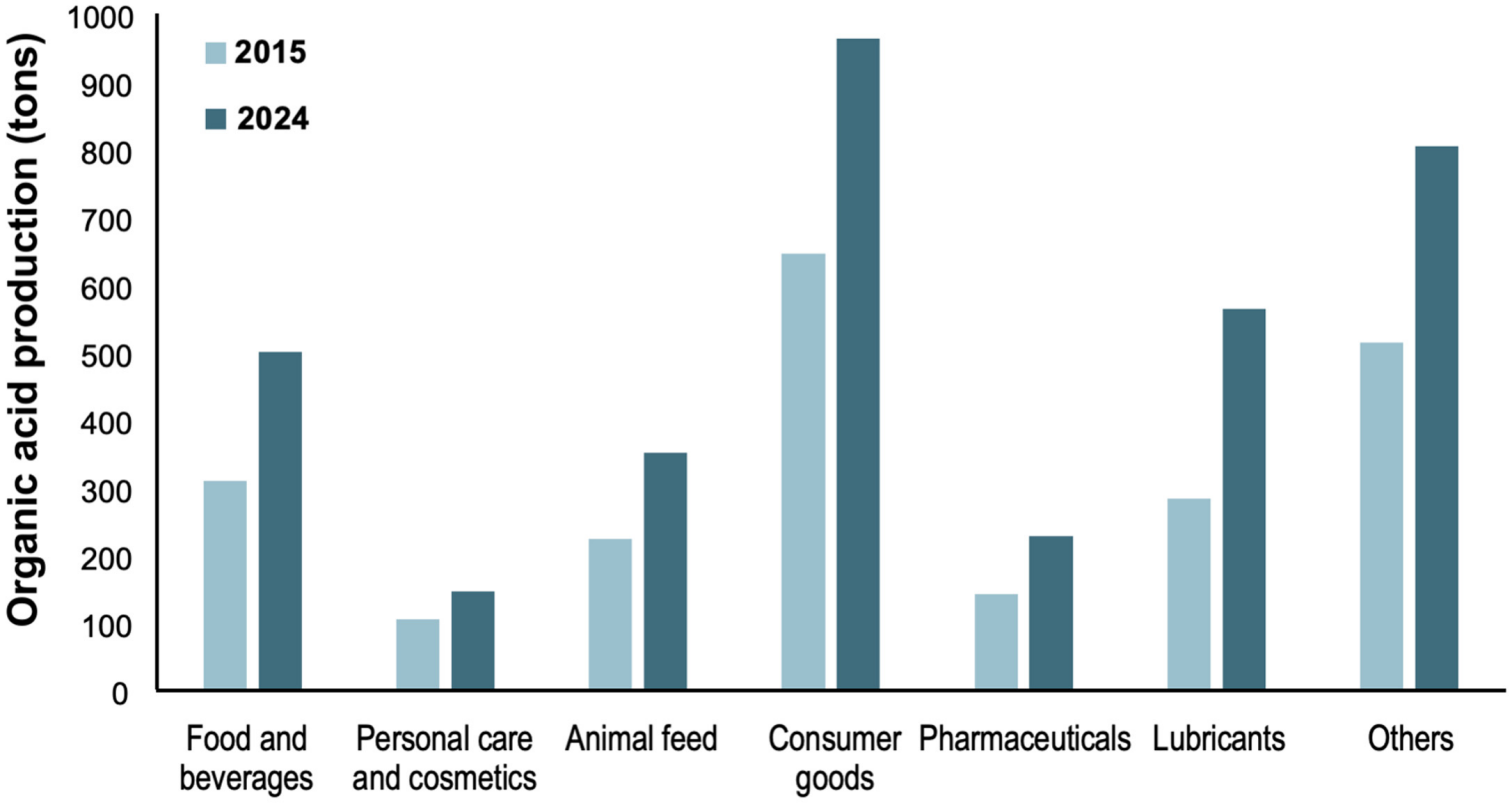
Annual production of organic acids according to market sector/application in 2015 and estimated growth for 2024 (adapted from https://www.alliedmarketresearch.com/organic-acids-market).

The global organic acids market was valued at 17 billion euros in 2016. The forecasts predict an annual growth of 8.3%, which should reach 30 billion euros by the year 2023 (Sahu 2017) with an impact in a broad range of industrial sectors (Fig. 1). The most significant contributions to this growth are the use of renewable resources, the rising market and the growing demand from developing countries for bio-based organic acids.

The industry of microbial organic acid production is under continuous development to increase cell factory productivity, yields and range of products. Along with classical strain engineering approaches and adaptive laboratory evolution (ALE) strategies, the development of recombinant DNA technology together with synthetic biology has allowed the rational engineering of organic acid-producing microbes. Meanwhile, beyond the classical industrial microbes, such as *Escherichia coli, Saccharomyces cerevisiae* and *Corynebacterium glutamicum*, we have witnessed the appearance of other species isolated from natural sources, displaying a high capacity to generate organic acids (Buschke *et al*. 2013; Na *et al*. 2012; Becker and Wittmann 2015).

### Membrane transporters as tools for the improvement of cell factories

Nowadays, most industrial microorganisms are metabolically engineered to produce specific products and/or to metabolize specific substrates. For decades, the transport mechanisms and energetics of these compounds were underestimated, and most attention was given to the engineering of metabolic pathways. Recently, the scientific community and biotech companies focused their efforts on transporter engineering, envisaging the development and improvement of microbial cell factories (Sauer *et al*. 2008; Boyarskiy and Tullman-Ercek 2015; Kell *et al*. 2015).

The microbial fermentation industry faces two major bottlenecks in the production line: the first relates to product accumulation and toxicity inside the cell and low product titers in the extracellular medium; the second is associated with cell factory capacity to assimilate carbon and energy sources for product biosynthesis. These two obstacles in microbial fermentation can be overcome by transport activity of endogenous or exogenous membrane transporters, importers and/or exporters, as well as their genetic manipulation regarding expression levels and generation of mutant alleles with increased transport capacity or altered specificity (Sauer *et al*. 2010; Boyarskiy and Tullman-Ercek 2015; Kell *et al*. 2015).

## MEMBRANE TRANSPORTERS IN THE IMPORT OF RENEWABLE SUBSTRATES

The most used renewable feedstocks in the bio-based industrial production of carboxylic acids are cheese whey, lignocellulosic biomass, glycerol and pectin-rich wastes (Alonso, Rendueles and Díaz 2015). Lactose is the most abundant sugar in cheese whey; xylose along with arabinose are abundant sugars in lignocellulosic hydrolysates; pectin-rich wastes, such as citrus and beet pulp, are rich in galacturonic acid (Alonso, Rendueles and Díaz 2015; Deng, Wang and Yan 2016). Frequently, these sugars are hardly assimilated and metabolized by microbial cell factories. This bottleneck is associated with the lack of membrane proteins or extracellular enzymes capable of respectively taking up or converting these substrates into assimilated forms. Therefore, membrane transporters are engineered in microbial cell factories to increase the efficiency of substrate influx, by altering transporter specificity, affinity and/or capacity, ultimately leading to improved production yields (Van Dyk 2008; Kell *et al*. 2015; Deng, Wang and Yan 2016).

Extensive efforts were devoted to the use of genetically engineered *E. coli* as a sustainable platform for the production of industrially important compounds, including organic acids (for a review see Chen *et al*. 2013; Yang *et al*. 2020). One of the few examples reporting the engineering of membrane transporters for the uptake of substrates to improve organic acid production in *E. coli* is the study by Wu, Liu and Singh (2018). Here, the increased production of catechol (gluconic acid precursor) was achieved after the co-expression of the catechol biosynthetic pathway and the transporter CouP, which enabled the uptake of aromatic compounds present in lignin. In a more recent work, Khunnonkwao *et al*. (2018) described the improvement of succinic acid production upon re-engineering of xylose transporters in *E. coli*. The filamentous fungus *Aspergillus niger* is the oldest industrial workhorse due to its great robustness to extreme acid environments and better fitness for industrial fermentation (Tong *et al*. 2019). Genome design and metabolic engineering approaches to optimize the *A. niger* cell factory for industrial citric acid production can be found in Tong *et al*. (2019). However, few transporter engineering strategies for substrate influx were described in this species. The endogenous low-affinity glucose transporter Lgt1 was expressed in the citrate-producing *A. niger* H915–1 strain, under the control of the low-pH-inducible promoter Pgas, leading to enhanced glucose absorption during the acid producing period and enhanced citrate production (Liu *et al*. 2018).

Yeasts are considered one of the most promising groups of industrial microorganisms to produce organic acids and ethanol. Thus hereafter, emphasis will be given on the functional expression of xylose, arabinose, lactose, glycerol and galacturonic acid transporters in attempts to improve organic acid biorefinery applications in yeasts.

### Xylose

The heterologous expression of xylose transporters in *S. cerevisiae* for the conversion of this lignocellulosic sugar into ethanol was extensively reported in the literature. More than 80 heterologous xylose transporters or putative xylose transporters have already been expressed in *S. cerevisiae* (for a review see Moysés *et al*. 2016). Significant examples include *SUT1*, *SUT2*, *XUT1*, *XUT3* (Xyp33), *XUT4*, Xyp29 (*STL12*), *SUT3* (Xyp37) from *Scheffersomyces stipitis*, *GXF1* from *Candida intermedia*, At5g59250 from *Arabidopsis thaliana*, An29–2 and An25 from *Neurospora crassa*, *xtrD* from *A. nidulans*, MgT05196 from *Meyerozyma guilliermondii* and *Xylh* from *Debaryomyces hansenii*. More than 80% of these putative transporters or annotated sugar transporters were not functional in *S. cerevisiae*, probably due to misfolding or improper localization (Moysés *et al*. 2016). The ones that were properly expressed in the HXT-null *S. cerevisiae* strain displayed activity for xylose transport, but the majority showed a preference for glucose over xylose.

### Arabinose

Along with xylose, arabinose is the second most abundant pentose sugar present in lignocellulosic hydrolysates. *S. cerevisiae* possesses endogenous arabinose transporters (Hxt9, Hxt10 and Gal2), Gal2 being the most prominent (Wang *et al*. 2013). However, the consumption of this sugar pentose is inefficient and inhibited by glucose, since Gal2 exhibits a much lower affinity for arabinose than for glucose or galactose (Becker and Boles 2003; Subtil and Boles 2011a). To improve the kinetics of arabinose uptake, transporter genes from other organisms have been functionally expressed in engineered arabinose-metabolizing *S. cerevisiae* strains. Two characterized L-arabinose transporters, LAT-1 from *N. crassa* and MtLAT-1 from *Myceliophthora thermophila* were expressed in this yeast (Li *et al*. 2015). The expression of both transporters in a *S. cerevisiae* strain containing a L-arabinose metabolic pathway resulted in a much faster L-arabinose utilization, greater biomass accumulation and higher ethanol production than the control strain. Expression of the *Pc*AraT arabinose transporter from *Penicillium chrysogenum* enabled growth on arabinose in the presence of glucose in a *S. cerevisiae* strain deficient in hexose phosphorylation and able to metabolize arabinose (Bracher *et al*. 2018). This transporter showed significantly higher affinity for arabinose compared to the endogenous Gal2 and had far less pronounced inhibition of arabinose uptake in the presence of glucose or xylose.

### Lactose

A recombinant *S. cerevisiae* flocculent strain heterologously expressing the β-galactosidase *LAC4* and lactose permease *LAC12* genes from *Kluyveromyces lactis* was used for ethanol production from lactose in a continuous culture operation (Domingues *et al*. 1999). This approach resulted in an ethanol production yield of 0.51 g/g of lactose. In continuous fermentation conditions, this engineered *S. cerevisiae* strain reached an ethanol productivity of 11 g/L/h, which represented a 7-fold rise compared with values of ethanol productivity from lactose previously mentioned in the literature (Domingues *et al*. 1999). The expression of the lactose transporter CDT-1, the intracellular β-galactosidase GH1–1 from *N. crassa* and the lactate dehydrogenase ldhA from *Rhizopus oryzae* in *S. cerevisiae* allowed the production of lactic acid from lactose, cow's milk, or whey (Turner *et al*. 2017). A lactic acid yield of 0.358 g/g lactose was achieved from a Yeast extract-Peptone medium containing about 80 g/L whey.

### Glycerol

Heterologous expression of glycerol facilitators (Fps1 homologs) from non-*Saccharomyces* yeast species that show superior growth on glycerol, e.g. *Cyberlindnera jadinii*, *Komagataella pastoris*, *Pachysolen tannophilus* and *Yarrowia lipolytica*, improved the maximum specific growth rates of the *S. cerevisiae* CBS 6412–13A strain by 30–40% in synthetic glycerol medium (Klein *et al*. 2016). Conversely, no improvement was visible after the overexpression of the endogenous *S. cerevisiae FPS1* gene. Deletion of the endogenous glycerol/H^+^ symporter *STL1* did not impair the superior growth of these strains. A significant increase in ethanol production (from none to 8.5 g/L) was obtained upon the expression of the heterologous aquaglyceroporin *CjFPS1* from *C. jadinii* in the strain CBS DHA, which catabolizes glycerol via the dihydroxyacetone (DHA) pathway(Asskamp, Klein and Nevoigt 2019). Further optimizations, including the reduction of oxygen availability in the shake flask cultures, increased the ethanol titer up to 15.7 g/L.

### Galacturonic acid

The introduction of the galacturonic acid transporter GAT1 from *N. crassa*, along with a fungal reductive pathway for galacturonic acid catabolism (*gaaA*, *gaaC* and *gaaD* from *A. niger* and *lgd1* from *Trichoderma reesei*), allowed the engineered *S. cerevisiae* strain to metabolize galacturonic acid (Biz *et al*. 2016). This strain was only able to catabolize galacturonic acid when a co-substrate was added (fructose). Tracing experiments with ^13^C-galacturonic acid revealed its conversion into glycerol (Biz *et al*. 2016). Recently, the expression of another galacturonic acid transporter, GatA from *A. niger*, allowed a more rapid consumption of this acid (Protzko *et al*. 2018). The involvement of endogenous yeast hexose transporters Hxt1–7 and Gal2 in the glucose-inhibited uptake of undissociated galacturonic acid in acidic conditions was also uncovered. Expression of glucose-insensitive GatA coupled with uronate dehydrogenase allowed the engineered *S. cerevisiae* strain to produce 8 g/L of meso-galactaric acid from citrus peel waste supplemented with additional glucose (Protzko *et al*. 2018).

## TRANSPORTER EXPRESSION FOR THE OPTIMIZATION OF ORGANIC ACID EFFLUX

Regarding the microbial production of organic acids, several reports pointed to the effectiveness and contribution of membrane transporters for product efflux. The organic acid transporters that thus far have been functionally characterized in yeast, fungi and bacteria are listed in Table 1. Among these transporters, a great majority belong to the 2-hydroxycarboxylate transporter (2-HCT) (TC 2.A.24), Divalent Anion:Na^+^ Symporter (DASS) (TC 2.A.47) and Sialate:H^+^ symporter (SHS) (TC 2.A.1.12) families. Members of 2-HCT are involved in the transport of di- and tricarboxylate substrates (malate/citrate uptake) with either Na^+^ or H^+^ as the co-substrate and precursor/product exchangers (Sobczak and Lolkema 2005). Some members mediate the transport of monocarboxylate substrates, 2-hydroxyisobutyrate and D-lactate (Bandell *et al*. 1998; Pudlik and Lolkema 2012). The integral membrane proteins of the DASS family are conserved from bacteria to humans. DASS proteins typically mediate the coupled uptake of Na^+^ ions and dicarboxylate, tricarboxylate, or sulfate (for a review see Lu 2019). A total of six members of DASS present a broad range of substrates from mono-, di- to tricarboxylates. The SHS transporter family, despite only having two distinct family members, the sialic acid transporter NanT, and the lactate/pyruvate:H^+^ symporter orthologues, is the one with most members characterized in yeast, accepting mainly mono- and dicarboxylates as well as sugar acids (Casal *et al*. 2008; Ribas *et al*. 2017). Next, we will highlight the transporters that had an impact on the improvement of cell factories.

**Table 1.** Microbial organic acid transporter proteins (experimentally verified). Table includes the transporter family, the species, the Transport Classification Database (TC number), the number of transmembrane segments (TMS), description of the transporter activity and references.

Family	Transporter protein	Species	TC number	TMS*	Description	References
2-HCT	CimH (YxkJ)	*Bacillus subtilis*	2.A.24.2.4	10	Electroneutral L-Malate/Citrate:H^+^ symporter; citrate (*K*_m_ 10 μM), L-Malate (*K*_m_ 1.5 mM)	Krom, Aardema and Lolkema (2001)
	MaeN (YufR)		2.A.24.2.3	11	Malate:Na^+^ symporter	Tanaka, Kobayashi and Ogasawara (2003)
	CitS	*Klebsiella pneumoniae*	2.A.24.1.1	12	Sodium:Citrate symporter	Kebbel *et al*. (2013)
	MleP	*Lactococcus lactis*	2.A.24	13	Sodium:Citrate symporter; malate (*K*_m_ 0.46 mM), lactate (*K*_m_ 4.6 mM)	Bandell *et al*. (1997); Poolman *et al*. (1991); Pudlik and Lolkema (2011)
	CitP (CitN)		2.A.24.3.1	12	Electrogenic citrate:L-Lactate exchanger; citrate (*K*_m_ 56 µM), malate (*K*_m_ 0.1 mM), lactate (*K*_m_ 26 mM)	Pudlik and Lolkema (2012)
	CitP	*Leuconostoc mesenteroides*	2.A.24.3.2	13	Citrate:Lactate antiporter; citrate, citramalate, malate, 2-Hydroxyisobutyrate and lactate	Bandell *et al*. (1998); Marty-Teysset *et al*. (1996)
AAEx	SucE	*Corynebacterium glutamicum*	2.A.81.1.3	9	Succinate exporter	Fukui *et al*. (2011)
AceTr	SatP	*Escherichia coli*	2.A.96.1.1	6 in hexameric channels	Acetate, lactate and succinate transporter; acetate (*K*_m_ 1.24 mM) and succinate (*K*_m_ 1.18 mM)	Sá-Pessoa *et al*. (2013)
	AceP	*Methanosarcina acetivorans*	2.A.96.1	6	Acetate transporter; acetate (*K*_m_ 0.49 mM)	Ribas *et al*. (2019)
	Ady2 (Ato1)	*Saccharomyces cerevisiae*	2.A.96.1.4	6	Acetate permease; acetate (*K*_m_ 0.84 mM), lactate, propionate and formate	Pacheco *et al*. (2012); Paiva *et al*. (2004); Ribas *et al*. (2019)
	Gpr1	*Yarrowia lipolytica*	2.A.96.1.2	6	Acetate transporter; acetate (*K*_m_ 0.95 mM)	Augstein *et al*.(2003); Paiva *et al*. (2004); Ribas *et al*. (2019)
Bestrophin	Best1 (AN2251)	*Aspergillus nidulans*	1.A.46.2.1	4	Ca^2+^-activated anion-selective channel; citrate, propionate, benzoate and sorbate	Galagan *et al*.(2005); Roberts, Milnes and Caddick (2011)
CitMHS	CitM	*Bacillus subtilis*	2.A.11.1.1	9	Citrate or D-Isocitrate divalent metal:H^+^ symporter; (*K*_m_ 35–63 µM), metal (in order of preference): Mg^2+^, Mn^2+^, Ni^2+^, Zn^2+^ and Co^2+^	Krom *et al*. (2000); Li and Pajor (2002)
	CitH (CitN)		2.A.11.1.2	11	Citrate divalent metal:H^+^ symporter: (*K*_m_ 35–63 µM) metal (in order of preference): Ca^2+^, Ba^2+^ and Sr^2+^	Krom *et al*. (2000)
	YRAO		2.A.11.1.5	13	Citrate:H^+^ symporter	Watanabe *et al*. (2012)
	CitH	*Corynebacterium glutamicum*	2.A.11.1.6	10	Divalent cation:citrate; citrate transport in complex with Ca^2+^ or Sr^2+^	Brocker *et al*. (2009)
DAACS	Dct	*Aspergillus carbonarius*	2.A.23.1.7	9	Fumarate, L-Aspartate: symporter	Yang *et al*. (2017)
		*Actinobacillus succinogenes*	2.A.23	12	Malate and citrate exporter	Darbani *et al*. (2019)
	DctA	*Bacillus subtilis*	2.A.23.1.6	8	Dicarboxylate:H^+^ symporter; succinate (*K*_m_ 2.6 μM), fumarate	Asai *et al*. (2000); Groeneveld *et al*. (2010)
		*Corynebacterium glutamicum*	2.A.23	7	Dicarboxylate:H^+^ symporter; L-Malate (*K*_m_ 736 μM), fumarate (*K*_m_ 232 μM), succinate (*K*_m_ 218 μM), oxaloacetate and glyoxylate	Youn *et al*. (2008)
		*Escherichia coli*	2.A.23.1.7	8	Aerobic dicarboxylate transporter; succinate (*K*_m_ 25 µM), orotate, fumarate and L- and D-Malate	Baker *et al*. (1996); Karinou *et al*. (2013); Kay and Kornberg (1971)
DASS	DccT (DcsT)	*Corynebacterium glutamicum*	2.A.47.1.12	14	Aerobic sodium dicarboxylate transporter; succinate (*K*_m_ 30 μM), fumarate (*K*_m_ 79 μM), malate (*K*_m_ 360 μM) and oxaloacetate	Ebbighausen, Weil and Krämer (1991); Teramoto *et al*. (2008); Youn *et al*. (2008)
	TtdT (YgjE)	*Escherichia coli*	2.A.47.3.3	12	L-Tartrate:Succinate antiporter; L-Tartrate (*K*_m_ 700 µM) uptake, succinate (*K*_m_ 400 µM) efflux	Kim and Unden (2007)
	CitT		2.A.47.3.2	13	Citrate:Succinate antiporter; citrate uptake and efflux of succinate, fumarate and tartrate.	Pos, Dimroth and Bott (1998)
	SLC13	*Actinobacillus succinogenes*	2.A.47	14	Citrate exporter	Darbani *et al*. (2019)
	SdcA		2.A.47.5.3	13	Dicarboxylate: Na^+^ transporter; fumarate (*K*_m_ 536 µM) and succinate (*K*_m_ 389 µM) uptake	Rhie *et al*. (2014)
	SdcS	*Staphylococcus aureus*	2.A.47.1.11	14	Dicarboxylate: Na^+^ symporter; succinate (*K*_m_ 7 mM), malate (*K*_m_ 8 mM), fumarate (*K*_m_ 15 mM), aspartate and α-Ketoglutarate transporter	Hall and Pajor (2005); Hall and Pajor (2007)
Dcu	DcuA	*Escherichia coli*	2.A.13.1.1	11	Anaerobic antiporter of aspartate, malate, fumarate and succinate; uptake and efflux of fumarate	Six *et al*. (1994); Zientz *et al*. (1996)
	DcuB		2.A.13.1.2	11	Anaerobic antiporter of aspartate, malate, fumarate and succinate; uptake and efflux of fumarate and citrate exporter	Darbani *et al*. (2019); Kim and Unden (2007); Six *et al*. (1994); Zientz *et al*. (1996)
DcuC	DcuC	*Escherichia coli*	2.A.61.1.1	12	Anaerobic electroneutral C4-dicarboxylate exchanger; dicarboxylate-proton symporter; citrate exporter	Chen *et al*. (2014); Darbani *et al*. (2019); Zientz *et al*. (1999,1996)
DHA1	CexA	*Aspergillus niger*	2.A.1.2	12	Citrate exporter	Steiger *et al*. (2019)
FNT	FocA	*Escherichia coli*	1.A.16.1.1	6 in pentameric channels (PDB 3KCU)	Exporter of acetate; (*K*_m_ 23.9 mM), lactate (*K*_m_ 96 mM) and pyruvate (*K*_m_ 11.6 mM); uptake/efflux of formate (*K*_m_ 11.7 mM)	Lü *et al*. (2012); Wang *et al*. (2009)
	PfFNT	*Plasmodium falciparum*	1.A.16.2.7	6	Lactate:H^+^ symporter; D-Lactate, pyruvate, acetate and formate	Marchetti *et al*. (2015); Wu *et al*.(2015)
LctP	LldP	*Escherichia coli*	2.A.14.1.1	12	Lactate permease; L-Lactate, D-Lactate and glycolate	Núñez *et al*. (2001)
	GlcA (YghK)		2.A.14.1.2	13	Glycolate permease; L-Lactate, D-Lactate and glycolate	Núñez *et al*. (2002); Núñez *et al*. (2001)
	LutP	*Bacillus subtilis*	2.A.14.1.3	14	Lactate permease	Chai, Kolter and Losick (2009)
		*Bacillus coagulans*	2.A.14.1	14	Lactate permease	Wang *et al*. (2019)
MFS	MfsA	*Aspergillus terreus*	2.A.1	12	Dicarboxylate transporter; Itaconate exporter	Hossain *et al*. (2016); Huang *et al*. (2014)
	Itp1	*Ustilago maydis*	2.A.1	12	Itaconate exporter	Geiser *et al*. (2016)
MHS	Dehp2	*Burkholderia caribensis*	2.A.1.6.11	12	Acetate/haloacid transporter; acetate, chloroacetate, bromoacetate, 2-chloropropionate; low-affinity to glycolate, lactate and pyruvate	Su *et al*. (2016); Su and Tsang (2013)
	Deh4p		2.A.1.6.13	11	Acetate/Monochloroacetate (haloacid) permease; acetate (*K*_m_ 5.5 μM) and monochloroacetate (*K*_m_ 9 μM)	Su *et al*. (2016); Su and Tsang (2013)
NhaC	MleN (YqkI)	*Bacillus subtilis*	2.A.35.1.2	10	Malate:Lactate antiporter coupled with proton uptake and sodium efflux; Malic^2−^-2H^+^: Na^+^-Lactate^1−^	Wei *et al*. (2000)
SHS	Jen1	*Saccharomyces cerevisiae*	2.A.1.12.2	12	Lactate/Pyruvate:H^+^ symporter; acetate (*K*_m_ 4.8 mM), lactate (*K*_m_ 0.2 mM), propionate, pyruvate (*K*_m_ 0.7 mM), selenite	Casal *et al*. (1999); McDermott, Rosen and Liu (2010); Soares-Silva *et al*. (2007, 2003)
	CaJen1	*Candida albicans*	2.A.1.12	10	Monocarboxylate permease; lactate (*K*_m_ 0.33 mM), pyruvate and propionate	Soares-Silva *et al*. (2004)
	CaJen2		2.A.1.12	10	Dicarboxylate permease; succinate (*K*_m_ 0.49 mM), malate (*K*_m_ 0.12 mM); affinity for the sugar acids gluconate, xylarate and mucate	Ribas *et al*. (2017); Vieira *et al*. (2010)
	DH17	*Debaryomyces hansenii*	2.A.1.12	12	Malate permease (*K*_m_ 0.27 mM)	Soares-Silva *et al*. (2015)
SHS	DH18	*Debaryomyces hansenii*	2.A.1.12	12	Succinate permease (*K*_m_ 0.31 mM)	Soares-Silva *et al*. (2015)
	DH24		2.A.1.12	12	Succinate permease (*K*_m_ 0.16 mM)	Soares-Silva *et al*. (2015)
	DH27		2.A.1.12	12	Acetate permease (*K*_m_ 0.94 mM)	Soares-Silva *et al*. (2015)
	KlJen1	*Kluyveromyces lactis*	2.A.1.12	12	Monocarboxylate permease; lactate (*K*_m_ 2.08 mM), pyruvate	Lodi *et al*. (2004); Queirós *et al*. (2007)
	KlJen2		2.A.1.12	11	Dicarboxylate permease; malate (*K*_m_ 0.15 mM), succinate (*K*_m_ 0.11 mM), fumarate; affinity for the sugar acids gluconate and saccharate	Lodi *et al*. (2004); Queirós *et al*. (2007); Ribas *et al*. (2019)
SSS	MctC	*Corynebacterium glutamicum*	2.A.21.7.3	13	Acetate/Propionate:H^+^ symporter; pyruvate (*K*_m_ 250 µM), acetate (*Km* 31 µM), propionate (*K*_m_ 9 µM)	Jolkver *et al*. (2009)
	ActP (YjcG)	*Escherichia coli*	2.A.21.7.2	13	Acetate (*K*_m_ 5.4 μM) and glyoxylate coupling ion: proton transporter, with affinity for tellurite	Elías *et al*. (2015); Gimenez *et al*. (2003)
	ActP1	*Rhodobacter capsulatus*	2.A.21.7.4	14	Acetate permease; acetate (*K*_m_ 1.89 mM), pyruvate, lactate, tellurite (*K*_m_ 163 μM)	Borghese *et al*. (2016); Borghese Cicerano and Zannoni (2011); Borghese and Zannoni (2010)
ST	KgtP (WitA)	*Escherichia coli*	2.A.1.6.2	12	α -Ketoglutarate (Oxoglutarate) :symporter; arabinose exporter	Koita and Rao (2012)
SulP	DauA (YchM)	*Escherichia coli*	2.A.53.3.11	11	Aerobic succinate transporter; succinate (*K*_m_ 0.56 mM), aspartate and fumarate	Karinou *et al*. (2013)
TDT	Mae1	*Schizosaccharomyces pombe*	2.A.16.2.1	10	Malate:H^+^ symporter; oxaloacetate, malonate, succinate, fumarate and thio-malate; exporter of fumarate, succinate and malate	Camarasa *et al*. (2001); Darbani *et al*. (2019); Grobler *et al*.(1995); Osawa and Matsumoto (2006)
	Ssu1	*Ustilago trichophora*	2.A.16	9	Malate transporter	Zambanini *et al*. (2017)
	Ssu2		2.A.16	9	Malate transporter	Zambanini *et al*. (2017)
TRAP-T	DctPQM	*Rhodobacter capsulatus*	2.A.56.1.1	12 (DctM) + 4 (DctQ) + receptor	Tripartite dicarboxylate: H^+^ symporter; malate (*K*_d_ 8.4 μM) competitively inhibited by fumarate (*K*_i_ 2 μM) and succinate (*K*_i_ 8 μM)	Forward *et al*. (1997)
TTT	TctABC	*Corynebacterium glutamicum*	2.A.80.1.4	12(TctA) + 4(TctB) + 1(TctC)	Citrate transport in complex with Ca^2+^ or Mg^2+^	Brocker *et al*. (2009)

* Number of TMS predicted with the TMHMM software (http://www.cbs.dtu.dk/services/TMHMM/) or verified.

TCDB Families: 2-HCT–2-Hydroxycarboxylate Transporter; AAEx—Aspartate:Alanine Exchanger; AceTr—Acetate Uptake Transporter; Bestrophin—Anion Channel-forming Bestrophin; CitMHS—Citrate-Mg^2+^:H^+^ (CitM) Citrate-Ca^2+^:H^+^ (CitH) Symporter; DAACS—Dicarboxylate/Amino Acid:Cation (Na^+^ or H^+^) Symporter; DASS—Divalent Anion:Na^+^ Symporter; Dcu—C4-Dicarboxylate Uptake; DcuC—C4-dicarboxylate Uptake C; DHA1–Drug:H^+^ Antiporter-1; FNT—Formate-Nitrite Transporter; LctP—Lactate Permease; MFS—Major Facilitator Superfamily; MHS—Metabolite:H^+^ Symporter; NhaC—Na^+^:H^+^ Antiporter; SHS—Sialate:H^+^ Symporter; SSS—Solute:Sodium Symporter; ST—Sugar transporter; SulP—Sulfate Permease; TDT—Telurite-resistance/Dicarboxylate Transporter; TRAP-T—Tripartite ATP-independent Periplasmic Transporter; TTT—Tripartite Tricarboxylate Transporter. na—not annotated at TC Database.

### Glutamic acid

The bacterium *Corynebacterium glutamicum* is used in microbial biotechnology for the production of glutamic acid. Glutamate efflux, triggered by increased mechanic tension, was associated with the activation of the channel NCgl1221 (MscCG; Nakamura *et al*. 2007; Becker *et al*. 2013), belonging to the MscS Family (TCDB 1.A.23 The Small Conductance Mechanosensitive Ion Channel). However, the activation mechanism of *C. glutamicum* mechanosensitive channels is not fully understood (for a review see Nakayama *et al*. 2019). Several channels of this family are described to play a critical role in product efflux of other amino acids, namely lysine, isoleucine, threonine, methionine and others (Van Dyk 2008; Kell *et al*. 2015).

### Malic acid

In the natural malic acid producer *Ustilago trichophora* RK089, the overexpression of two endogenous malate transporter genes improved the production yields by 54% (Zambanini *et al*. 2017). The overexpression of pyruvate carboxylase (*pyc*) together with two malate dehydrogenases (*mdh1*, *mdh2*), and two malate transporters (*ssu1, ssu2*) was carried out in a laboratory-evolved *U. trichophora* strain that reached an extracellular malate titer of 120 g/L. Wild-type *S. cerevisiae* strains produce low levels of malate. High yield production of malic acid required the elimination of alcoholic fermentation, which in this yeast occurs under fully aerobic conditions when high concentrations of sugar are present (Zelle *et al*. 2008). The metabolic engineering of a *S. cerevisiae* strain allowed an increase up to 10-fold of malic acid titer relative to the control strain (Zelle *et al*. 2008). This was achieved through the engineering of a glucose-tolerant, C_2_-independent pyruvate decarboxylase-negative strain, together with: (i) the overexpression of the endogenous pyruvate carboxylase encoded by PYC2, (ii) the overexpression of an allele of the peroxisomal malate dehydrogenase MDH3 gene targeted to the cytoplasm and (iii) the functional expression of the *S. pombe* malate transporter *Sp*Mae1. These modifications *per se* improved malate production, and the combination of all genetic modifications reached a malate titer of approximately 59 g/L(Zelle *et al*. 2008). Recently, seven dicarboxylic acid transporters were expressed in a *S. cerevisiae* strain engineered for dicarboxylic acid production (Darbani *et al*. 2019). In this work, the expression of the *Sp*Mae1 homologous gene from *Aspergillus carbonarius*, AcDct, increased malate titer up to 12-fold. Upon *Sp*Mae1 expression, the following titers were obtained for malate (8 fold-4.3 g/L), succinate (3 fold-2.6 g/L) and fumarate (5 fold-0.33 g/L).

### Fumaric acid

The overexpression of the *S. cerevisiae* mitochondrial succinate-fumarate carrier *SFC1* gene enhanced fumarate export and production by 47.6% in this yeast (Xu *et al*. 2012). A *S. cerevisiae* strain engineered for the production of fumarate, deleted in the fumarase *FUM1* gene and expressing the RoPYC pyruvate carboxylase gene of *R. oryzae* and the endogenous *SFC1* gene, resulted in a titer of 1.7 g/L of fumarate in batch culture.

Using a different approach, fumarate production in *Candida glabrata* was improved by overexpressing the Sfc1 mitochondrial carrier in combination with the heterologous expression of *Sp*Mae1 (Chen *et al*. 2015). This work established the metabolic engineering of the tricarboxylic acid cycle in *C. glabrata* to construct the oxidative pathway for fumarate production. Thus, a set of genetic modifications to manipulate the oxidative pathway was applied in the α-ketoglutarate dehydrogenase complex, succinyl-CoA synthetase and succinate dehydrogenase. As a result, the *C. glabrata* producer strain reached a fumarate titer of 8.24 g/L. Overexpression of the argininosuccinate lyase gene led to a fumarate increase up to 9.96 g/L. The additional expression of two dicarboxylic acid transporters, Sfc1 and *Sp*Mae1, allowed an improvement of fumarate production (15.76 g/L; Chen *et al*. 2015).

In *E. coli* a set of C_4_-dicarboxylate transporters from different organisms were cloned in a fumaric acid-producing strain deleted in the genes *fumABC*, *frdABCD*, *iclR* and *arcA*, to evaluate their impact on the production of this acid (Zhang *et al*. 2017b). It was the overexpression of the endogenous transporters *DcuB*, an anaerobic fumarate–succinate antiporter (Zientz, Six and Unden 1996), and *DcuC*, a C_4_-dicarboxylate carrier that promotes succinate efflux during glucose fermentation (Zientz *et al*. 1999), that displayed the highest impact on the production of fumaric acid. These lead to an increase of fumaric acid yield by 48.5% and 53.1%, respectively. In fed-batch fermentation culture, the fumaric acid producer strain overexpressing the *dcuB* gene reached 9.42 g/L of fumaric acid after 50 h (Zhang *et al*. 2017b).

### Succinic acid

Modulation of the simultaneous expression of *E. coli* transporter genes *dcuB* and *dcuC* led to a 34% increase of succinic acid titer in an engineered *E. coli* strain (Chen *et al*. 2014). In this work, four *E. coli* Dcu C_4_-dicarboxylate transporters were exploited for succinate export. Single deletion of *dcuA* or *dcuD* did not affect the export of this organic acid, while *dcuB* and *dcuC* deletion led to 15% and 11% decrease of succinate extracellular titer, respectively. The combined deletion of *dcuB* and *dcuC* genes resulted in a 90% decrease of succinate titer. As a result, a ribosome binding site library was investigated to modulate and increase the co-expression of *dcuB* and *dcuC*, which led to a 34% increase of succinate titer produced by *E. coli* (Chen *et al*. 2014).

In *C. glutamicum*, the overexpression of the endogenous succinate exporter, SucE, increased succinate yield in an engineered strain (Zhu *et al*. 2014). A dual-route for anaerobic succinate production was devised, involving the reconstruction of the glyoxylate pathway by overexpressing isocitrate lyase, malate synthase and citrate synthase. This succinate producer strain reached a succinate yield of 1.34 mol/mol of glucose. The additional overexpression of the endogenous succinate exporter, SucE, increased succinate yield to 1.43 mol/mol of glucose. In anaerobic fed-batch fermentation, the *C. glutamicum* succinate producer strain overexpressing SucE led to a titer of 109 g/L succinate.

### Itaconic acid

Expression of two *Aspergillus terreus* genes encoding organic acid transporters, *mttA* and *mfsA*, increased itaconic acid production in an *A. niger* strain expressing the cis-aconitate decarboxylase (Li *et al*. 2011, 2012). MttA is a mitochondrial tricarboxylic acid transporter that preferentially transports cis-aconitate instead of citrate (Steiger *et al*. 2016). MfsA is an itaconate plasma membrane exporter (Huang *et al*. 2014; Hossain *et al*. 2016). The strains expressing *mttA* or *mfsA* displayed an increased itaconic acid (1.5 g/L) production when compared with an *A. niger* strain expressing only cis-aconitate decarboxylase (0.8 g/L; Li *et al*. 2013). Interestingly, the production did not increase further when both transporters were co-expressed (0.9 g/L). In a previous study in *A. terreus*, the overexpression of a bacterial hemoglobin (vgb) led to an increased dissolved oxygen level, having a strong effect on itaconic acid production (Lin *et al*. 2004). Additional optimization was achieved by overexpression of the fungal hemoglobin domain *hbd1* and deletion of the oxaloacetate acetylhydrolase *oahA* gene, in combination with controlled batch fermentation conditions, resulting in the increase of the production level from 0.8 to 2.5 g/L of itaconic acid (Li *et al*. 2013). In a subsequent study, a titer of 26.2 g/L and a maximum production rate of 0.35 g/L/h were reached by overexpressing the cytosolic citrate synthase *citB* (Hossain *et al*. 2016).

### Lactic acid

The *S. cerevisiae* genome encodes at least two plasma membrane monocarboxylate transporters, Jen1 (Casal *et al*. 1999; Soares-Silva *et al*. 2003) and Ady2 (Paiva *et al*. 2004; Ribas *et al*. 2019) with distinct specificities, mode of action and regulation mechanisms (Casal *et al*. 2008, 2016). In a *S. cerevisiae* strain engineered for lactate production, the constitutive expression of these two transporters resulted in a higher accumulation of lactic acid in the extracellular medium (Pacheco *et al*. 2012). In this study, the authors expressed the lactate-dehydrogenase *LDH* gene from *L. casei* in the *S. cerevisiae* W303–1A parental strain and in the three isogenic strains *jen1∆*, *ady2∆* and *jen1∆ ady2*∆ to allow lactate production. All the deleted strains expressing *LDH* were able to produce higher titers of lactic acid compared with the parental isogenic strain. Moreover, the constitutive expression of *JEN1* or *ADY2* genes, along with *LDH*, resulted in the higher external accumulation of lactic acid in the presence of glucose. Upon glucose depletion, lactate consumption was also more pronounced in cells expressing Jen1 and/or Ady2, suggesting the involvement of these transporters in both the import and export of lactic acid (Pacheco *et al*. 2012).

### Citric acid

In a recent work, Steiger *et al*. (2019) identified CexA, the longtime sought citrate exporter from *A. niger*. The constitutive and inducible overexpression of CexA in the native citric acid-producing species *A. niger*, resulted in significant increases in secreted citric acid (Steiger *et al*. 2019). The inducible system reached 109 g/L citric acid, five times higher than the parental wild-type strain and three times higher than the constitutive expression system.

## ENGINEERING MEMBRANE TRANSPORTERS

Membrane transporters, like any protein, can display substrate promiscuity, altered conformation, distinct affinity and capacity depending on physiological conditions, as well as alterations in folding and stability. Finding a membrane transporter, either for import or export, might not be enough to achieve the levels of cell factory productivity needed to obtain a cost-effective and sustainable bioproduction process. The engineering of membrane proteins can diminish these constraints by tuning the activity towards specific conditions and substrates. This approach is frequently achieved by ALE experiments, mutagenesis or recombination involving methods of synthetic biology (Van Dyk 2008; Kell *et al*. 2015; Moysés *et al*. 2016). ALE of host organisms combined with the identification of responsible genetic changes and subsequent reverse engineering, is a powerful approach to obtain novel or improved substrate specificity of membrane transporters.

### Engineering sugar transporters

Whereas it is common to look for exogenous transporters to be cloned into producer strains, the endogenous transportome can be used as a pool of transporters for cell factory optimization. One such example is the complex landscape of the *S. cerevisiae* genome that includes 20 transporter proteins belonging to the Hexose Transporter (HXT) Family, with great potential to be exploited in cell factories for the uptake of renewable sugars from lignocellulosic wastes (Kruckeberg 1996; Moysés *et al*. 2016). By using molecular modeling and docking studies, the endogenous *S. cerevisiae* Gal2 transporter was engineered to improve L-arabinose transport capacity (Subtil and Boles 2011b). In this study, nine residues were found to interact with L-arabinose. Rational protein design by directed mutagenesis allowed an increase of transporter capacity for L-arabinose. Besides the gain of function associated with arabinose transport capacity, the F85S mutation specifically improved xylose transport (Wang *et al*. 2017). In another study, the combination of computer-assisted modeling, site-directed mutagenesis, error-prone PCR approaches and selective growth conditions, resulted in the identification of residues in both Hxt7 and Gal2 that yielded glucose-insensitive xylose transporters (Farwick *et al*. 2014). The mutant Gal2-N376F had the highest affinity for D-xylose, along with a moderate transport rate for this pentose sugar, and completely lost the ability to transport hexoses (Farwick *et al*. 2014).

To obtain a transporter able to sufficiently import arabinose in the presence of glucose and xylose, a strain deficient in glucose phosphorylation and able to metabolize arabinose was created (Verhoeven *et al*. 2018). Subsequently, the engineered strain was grown in medium with these three sugars. This way, conditions were met where arabinose became the only metabolizable sugar within the media, while glucose and xylose were exerting selective pressure towards the evolution of an arabinose transporter uninhibited by glucose and xylose. Consequently, mutations within hexose transporter Gal2 in residues T89 and N376 were found to significantly increase the *K*_m_ value of Gal2 for glucose, and decrease the *K*_m_ value for arabinose, enabling superior growth of the engineered strain in a medium containing the three sugars (Verhoeven *et al*. 2018).

### Engineering organic acid transporters

In an attempt to evolve an efficient fumarate exporter in *S. cerevisiae*, a knock-out strategy was implemented in which fumaric acid was turned into the energetically more favorable catabolic product, by deletion of the fumarase (*FUM1*) and glucose 6-phosphate dehydrogenase (*ZWF1*) genes (Shah 2016). The malate and succinate transporter DCT_02 from *A. niger* was used as a template expected to evolve into an efficient fumarate exporter. However, the evolution experiment did not yield the desired results, since only malate and succinate were secreted to the extracellular medium, and further strategy refinement is required (Shah 2016).

Through a rationally designed site-directed mutagenesis strategy, the substrate specificity of the yeast Jen1 monocarboxylate transporter was altered to acquire the ability to transport the dicarboxylic acids succinate (F270A and F270G; Soares-Silva *et al*. 2011) and saccharate (S271Q; Ribas *et al*. 2017).

In two independent evolution experiments, *S. cerevisiae* strains deficient in Jen1 were evolved for growth on lactate as sole carbon and energy source (de Kok *et al*. 2012). Whole-genome resequencing of evolved strains uncovered the presence of single nucleotide changes in the acetate transporter gene *ADY2* (C755G/L219V and C655G/A252G). These Ady2 mutated alleles encode efficient lactate transporters.

Presently, new relevant roles of protein transporters are being uncovered, namely at the level of improving industrial strain's tolerance to by-products. An example of the complexity of the roles of transporters in regulatory networks is reported by Zang *et al*. (2017a) who, in the presence of 3.6 g/L acetic acid pH 3.7, observed an increment of 14.7% in the final ethanol concentration for the *S. cerevisiae *strain lacking the *ADY2* gene. By impairing acetate uptake from the extracellular space, the accumulation of intracellular acetate was reduced, and as consequence cells acquired increased tolerance towards this organic acid.

## FUTURE PERSPECTIVES FOR TRANSPORT ENGINEERING

It is expected that in the near future, biorefineries increase the production of platform chemicals from renewable resources (Takkellapati, Li and Gonzalez 2018). The exploitation of industrial biowastes to sustain microbial cell growth and valorize biomass conversion into organic acids is one of these current trends. Achieving optimal processes requires industrially robust strains. One of the major bottlenecks for the efficient and cost-effective bioproduction of organic acids is their export through the microbial plasma membrane. Membrane transporter proteins are thus crucial elements for the optimization of this process. In this review, we presented examples of the most relevant and emerging cell factories for the production of organic acids, as well as the engineering strategies used to turn them into efficient producers of this family of compounds.

**Figure 2.**
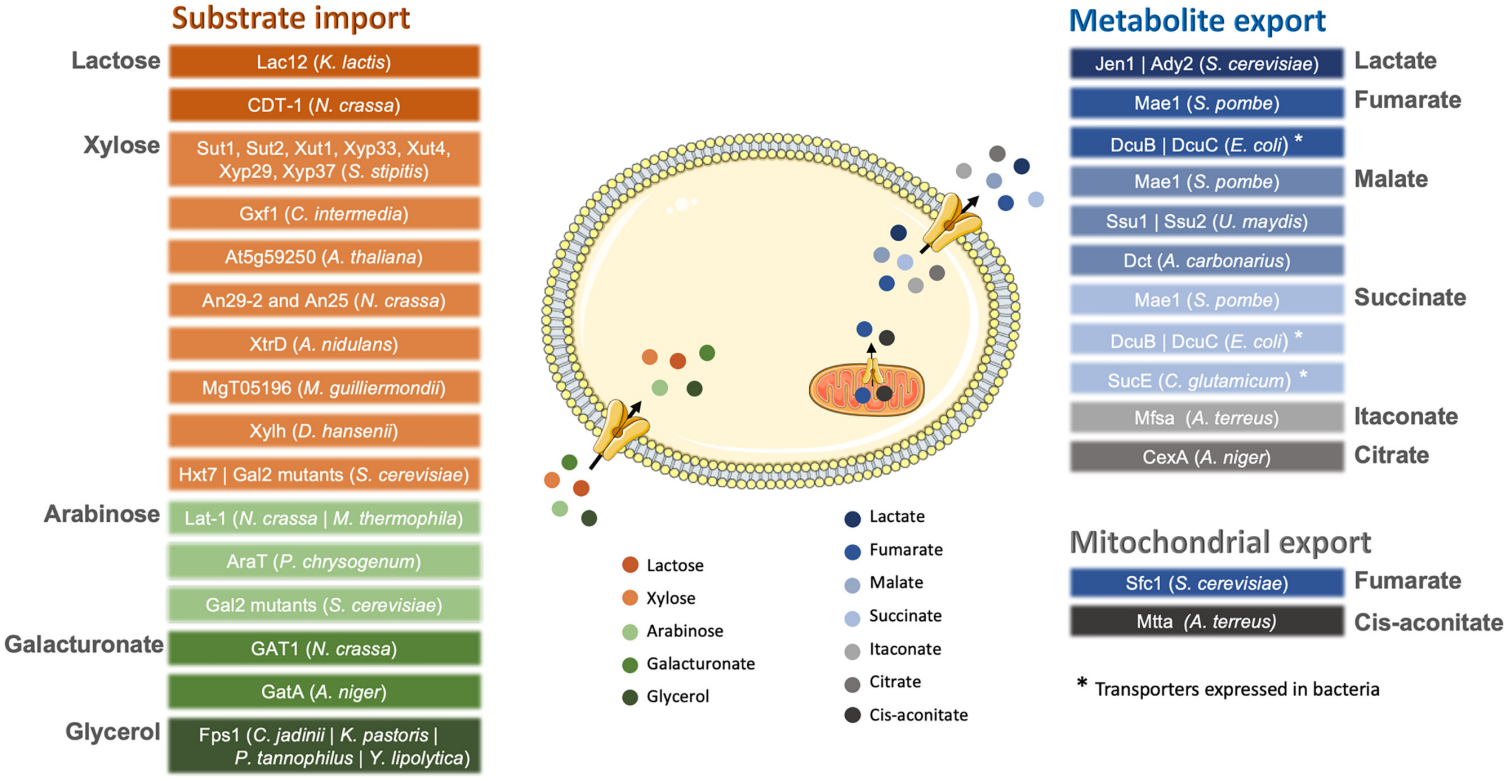
The expression of endogenous or exogenous membrane transporter genes in engineered bacteria, yeast and filamentous fungi, allows the uptake of renewable substrates, as well as the export and extracellular accumulation of specialty organic acids. Transporters on the left were expressed in the plasma membrane, to promote the import of substrates. The transporters on the right were expressed either in the inner mitochondrial membrane or in the plasma membrane, to promote the export of organic acids. The black arrows indicate the direction of the transport, either to the cytoplasm, out of the mitochondria or to the extracellular medium. Transporters expressed in bacteria are marked with *. The figure was produced using the vector image bank of Servier Medical Art (http://smart.servier.com/).

In recent years, a great effort was dedicated to transporter engineering, envisaging the development and improvement of microbial cell factories. Examples of transporters engineered in producer strains, especially in the yeast *S. cerevisiae*, are summarized in Fig. 2. Despite these advances, as the functional and structural characterization of membrane proteins is still a cumbersome process, the redesigning and engineering of optimized cell membrane transporters for industrial organic acid production is still at an early stage (Boyarskiy and Tullman-Ercek 2015; Kell *et al*. 2015). Different strategies can be followed to obtain improved transporters, namely with higher activity, altered substrate specificity and product selectivity. ALE can be a suitable approach when the desired transport process is directly linked to a selective advantage, such as the import of a sole carbon source necessary for growth. Nevertheless, its employment to generate improved transporters for the efflux of solutes can represent a demanding challenge. The complexity of biorefineries relies on many factors, including the optimization of several transporters, with complementary kinetic and regulatory properties (Verhoeven *et al*. 2018). Structure-based or computer simulation-based protein engineering is a powerful approach. However, these methods are hampered by the low number of robust three-dimensional structural models of transporter proteins. According to the Protein Data Bank (https://www.rcsb.org), transporter 3D structures account for less than 10% of the total database entries, showing that membrane proteins remain until now mostly uncharacterized, which evidences the need to increase the existing knowledge on this field. The recent identification of the gene encoding the long-sought citrate exporter from *A. niger* (Odoni *et al*. 2019; Steiger *et al*. 2019), is an example of the effort that must be carried out towards the identification of new transporters.

The biodiversity of the microbial world is an excellent pool to uncover relevant transporters for organic acid production. Achieving the efficient heterologous expression of transporters is crucial to improve the robustness of microbial cell factories. For instance, the proper expression of bacterial transporters in fungi is constrained due to membrane incompatibility, low expression levels and folding difficulties (Young *et al*. 2011), limiting the options for prokaryotic transporter expression in eukaryotic cells. Still, the versatility and plasticity of membrane transporters suggest a promising future towards the optimization and implementation of platform chemical bioproduction at the industrial scale.
